# Association between transcranial direct current stimulation and disability and quality of life in individuals with Parkinsonism: cross-sectional study

**DOI:** 10.3389/fneur.2025.1601778

**Published:** 2025-05-21

**Authors:** Ravi Shankar Reddy, Jaya Shanker Tedla, Irshad Ahmad, Venkata Nagaraj Kakaraparthi, Snehil Dixit, Kumar Gular, Paul Silvian Samuel, Suhail Mansour Aljehani, Feras Ahmed Alarabi

**Affiliations:** ^1^Program of Physical Therapy, Department of Medical Rehabilitation Sciences, College of Applied Medical Sciences, King Khalid University, Abha, Saudi Arabia; ^2^King Salman Center for Disability Research, Riyadh, Saudi Arabia

**Keywords:** Parkinsonism, tDCS, disability, quality of life, neuromodulation

## Abstract

**Background:**

Parkinsonism is a progressive neurodegenerative disorder characterized by motor and non-motor impairments, significantly impacting quality of life (QoL). Transcranial direct current stimulation (tDCS) has shown promise in improving motor and cognitive functions when combined with physical therapy. This study aimed to explore the association between tDCS exposure and disability levels, as well as its impact on self-reported QoL in individuals with Parkinsonism undergoing physical therapy.

**Methods:**

This cross-sectional study enrolled 51 participants diagnosed with Parkinsonism from a tertiary care hospital’s neurology outpatient clinic. Based on clinical records of tDCS sessions, participants were stratified into tDCS-exposed and non-exposed groups. Disability was assessed using the World Health Organization Disability Assessment Schedule, and QoL was measured using the Parkinson’s Disease Questionnaire (PDQ-39). Statistical analyses included *t*-tests for comparing means and Pearson correlation coefficients for assessing relationships between tDCS exposure, disability, and QoL.

**Results:**

The tDCS-exposed group demonstrated lower mean disability scores (WHODAS 2.0: 42.50 ± 8.12) and better quality of life scores (PDQ-39: 35.10 ± 6.45) compared to the non-exposed group (WHODAS 2.0: 45.30 ± 9.21; PDQ-39: 40.15 ± 7.32); however, these differences were not statistically significant (disability: *p* = 0.131; QoL: *p* = 0.236). Subgroup analyses revealed statistically significant improvements among participants under 65 years of age (disability mean difference = −3.3, 95% CI: −6.17 to −0.43, *p* = 0.023) and those in Hoehn and Yahr stages 1–2 (QoL mean difference = −3.7, 95% CI: −6.16 to −1.24, *p* = 0.004). Additionally, a moderate negative correlation was observed between tDCS session frequency and disability scores (*r* = −0.60, 95% CI: −0.78 to −0.30, *p* = 0.04), and a weak negative correlation with quality of life scores (*r* = −0.43, 95% CI: −0.66 to −0.11, *p* = 0.039).

**Conclusion:**

These findings suggest possible associations between tDCS exposure and clinical outcomes in individuals with Parkinsonism; however, due to the cross-sectional design and underpowered subgroup analyses, results should be interpreted with caution and viewed as hypothesis-generating.

## 1 Introduction

Parkinsonism encompasses a range of neurodegenerative conditions characterized by motor and non-motor symptoms, primarily caused by the loss of dopaminergic neurons in the basal ganglia (1). Key motor symptoms include bradykinesia, rigidity, resting tremor, and postural instability, which typically worsen over time, leading to significant disability and reduced quality of life (2). Despite advancements in medications and surgeries, their long-term effectiveness can diminish over time and may be accompanied by adverse effects (3). As a result, there is growing interest in complementary treatments such as transcranial direct current stimulation (tDCS), a non-invasive brain stimulation technique. tDCS applies a mild electrical current to modify neuronal activity and enhance neuroplasticity, showing promise in alleviating both motor and cognitive impairments associated with Parkinsonism and other neurological disorders (4).

The relationship between tDCS and disability levels in Parkinsonism has been an area of growing interest, particularly in the context of physical therapy interventions (5). Physical therapy is a cornerstone of non-pharmacological management, aiming to enhance motor function, balance, and mobility (6). However, its effectiveness can be hindered by neurodegeneration and limited neuroplasticity (7). By modulating cortical excitability and facilitating synaptic remodeling, tDCS may enhance motor performance and functional independence when combined with physical therapy (8). However, there is limited evidence on the specific impact of tDCS on disability levels in individuals with Parkinsonism, warranting further investigation to elucidate its therapeutic potential in this domain.

Quality of life is profoundly affected in Parkinsonism, not only due to motor impairments but also because of non-motor symptoms such as fatigue, mood disorders, and reduced social participation (9). Self-reported quality of life measures, particularly in domains like mobility, daily activities, and social interactions, provide valuable insights into the holistic impact of the disease (10). tDCS, by targeting both motor and non-motor cortical areas, holds promise in improving quality of life by alleviating physical and cognitive symptoms (11). While existing research has primarily focused on motor outcomes, the broader implications of tDCS on psychosocial dimensions of quality of life remain underexplored (12). Understanding these effects is crucial to developing comprehensive care strategies that address the multifaceted needs of individuals with Parkinsonism.

The need for this study arises from a significant gap in the current literature regarding the role of tDCS in reducing disability and enhancing quality of life in Parkinsonism. Most existing studies have focused on short-term motor improvements or isolated outcomes, often without considering the synergistic effects of tDCS with physical therapy (13, 14). Moreover, limited research explores how patient characteristics, such as age and disease stage, influence the efficacy of tDCS, restricting the ability to optimize its interventions (15). By examining both disability levels and quality of life outcomes, this study aims to provide a more comprehensive understanding of the associations between tDCS exposure and clinical outcomes in Parkinsonism. The objectives of this study are twofold: first, to assess the association between the use of tDCS and levels of disability in individuals with Parkinsonism undergoing physical therapy, and second, to examine the association between tDCS exposure and self-reported quality of life, focusing on mobility, daily activities, and social participation.

## 2 Methods

### 2.1 Study site, study design, and ethics

In this cross-sectional analytical study, data on the use of tDCS and its corresponding clinical outcomes were gathered concurrently from August 2023 to May 2024 at the Neurology Department of King Khalid University clinics. The research protocol received approval from the Institutional Ethics Committee (ECM#2023–3,304).

### 2.2 Participants

The study enrolled patients diagnosed with Parkinsonism from the outpatient neurology clinic of KKU-affiliated hospitals, a tertiary care teaching hospital, from April 2023 to March 2024. The diagnosis was based on the United Kingdom Parkinson’s Disease Society Brain Bank criteria and confirmed by a certified neurologist (16). Participants meeting inclusion criteria, aged 40–80 years with confirmed Parkinsonism for at least 1 year and Hoehn and Yahr (H&Y) stages 1–4 (17), were stratified into two groups based on their exposure to tDCS. Session details were documented in clinical records. In addition to regular physical therapy (≥3 sessions/week), eligible participants were required to provide written informed consent. Exclusion criteria included severe cognitive impairment (Montreal Cognitive Assessment (MoCA) < 26) and concurrent neurological or psychiatric conditions that could potentially confound the outcomes. A consecutive sampling method was used to recruit participants; all eligible participants visiting the clinic during the study period were invited to participate. Individuals satisfying the inclusion criteria received thorough baseline assessments of demographics and clinical features.

### 2.3 Variables

The primary outcomes assessed in this study included disability levels and quality of life, evaluated using established measurement tools. Disability was measured using the World Health Organization Disability Assessment Schedule 2.0 (WHODAS 2.0) (18), which assesses functioning across six domains: cognition, mobility, self-care, interpersonal relationships, life activities, and participation. Scores were computed following WHODAS 2.0 guidelines, where higher scores indicate greater disability levels. Quality of life was assessed with the Parkinson’s Disease Questionnaire (PDQ-39) (19), which evaluates eight domains pertinent to Parkinsonism, such as mobility, daily activities, emotional well-being, and social participation. Higher scores on the PDQ-39 indicate poorer quality of life across both specific domains and overall assessment.

The independent variable in this study was tDCS exposure, classified into two groups: ‘tDCS users’ and ‘non-users.’ Additionally, to explore further, the number of tDCS sessions completed before assessing outcomes was recorded from clinical records. tDCS was administered using a bipolar direct current stimulator (Model: Soterix Medical 1×1 tDCS device) (19), delivering a constant current of 2 mA through saline-soaked sponge electrodes sized 5 × 7 cm. The anodal electrode was positioned over the primary motor cortex (M1) of the dominant hemisphere, determined using the 10–20 EEG system (at C3 or C4 based on handedness), while the cathodal electrode was placed over the contralateral supraorbital region. Each tDCS session lasted 20 min and was conducted thrice weekly over 4 weeks, totaling 20 sessions. Stimulation was delivered in a ramp-up mode, gradually increasing to the target intensity over the first 30 s to minimize discomfort. Participants remained relaxed during stimulation and were monitored for adverse effects, including tingling, headache, and skin irritation. Adherence was tracked through a stimulation log maintained by the research team. This stimulation protocol was selected based on prior research demonstrating enhanced motor function and neuroplasticity in Parkinsonism with M1-targeted anodal stimulation. The 2-mA intensity and 20-min duration were chosen to optimize cortical excitability modulation while ensuring safety and tolerability, consistent with established tDCS guidelines in neurorehabilitation. Adherence to the full 20-session protocol was monitored using a stimulation log maintained by the research team. Participants in the tDCS group completed a mean of 10.18 ± 2.45 sessions, with adherence rates recorded and reviewed at each follow-up. All outcome assessments were conducted within 1 week of the final tDCS session as part of a single-point data collection protocol; no extended follow-up period was included in this cross-sectional study.

Participants in both the tDCS and non-tDCS groups received a standardized supervised physical therapy (PT) program tailored for individuals with Parkinsonism. The PT sessions were conducted three to five times per week, each lasting 45–60 min, and were supervised by licensed physical therapists specializing in neurorehabilitation. The sessions followed a structured regimen designed to address motor impairments and functional limitations commonly associated with Parkinsonism. The PT program incorporated gait training, focusing on stride length, step symmetry, and turning strategies to improve ambulation and reduce freezing episodes; balance and postural stability exercises, including weight-shifting drills, single-leg stance exercises, and perturbation training to enhance equilibrium and minimize fall risk; and lower and upper limb strengthening, utilizing body weight, resistance bands, and light weights to maintain muscle strength and prevent sarcopenia. Additionally, task-specific training was incorporated, emphasizing functional movements such as sit-to-stand transitions, stair climbing, and dual-task exercises to improve real-world mobility. Flexibility exercises targeting rigidity-prone areas such as the hip flexors, hamstrings, and paraspinal muscles were included to enhance range of motion and counteract the stiffness associated with Parkinsonism. The program also integrated respiratory exercises to support breathing control and endurance. All sessions followed a progressive approach, adjusting intensity and complexity based on individual performance and functional capacity. The PT regimen was standardized across participants, ensuring consistency while allowing for minor modifications based on disease severity (Hoehn & Yahr stage) and individual needs. Adherence to the program was monitored through session attendance logs, and participants were encouraged to perform home-based exercises to reinforce therapy benefits.

Cognitive function was evaluated using the Montreal Cognitive Assessment (MoCA) to account for potential cognitive impairment (20). Sleep quality was assessed with the Pittsburgh Sleep Quality Index (PSQI) (21), a self-reported questionnaire that examines sleep disturbances, sleep latency, and overall sleep quality. Fatigue levels were measured using the Fatigue Severity Scale (FSS), which assesses the impact of fatigue on daily activities (22). Medication adherence was self-reported by participants and computed as the percentage of prescribed doses taken during the study period.

### 2.4 Data analysis

Group comparisons for continuous variables, such as disability scores (WHODAS 2.0), quality of life scores (PDQ-39), and other clinical parameters, were conducted using independent samples *t*-tests. Subgroup analyses by age and disease stage were also performed using *t*-tests. Pearson’s correlation coefficients (r) were calculated to evaluate the strength and direction of associations between tDCS frequency and clinical outcomes, such as disability, quality of life, physical therapy frequency, and daily step count. Multiple linear regression analyses were conducted to examine the association between tDCS exposure and clinical outcomes, adjusting for covariates such as age, gender, disease stage, and physical therapy frequency. Given the cross-sectional design, these analyses were used to identify associations rather than causal relationships. Assumptions for parametric tests, including normality, homogeneity of variance, and independence, were assessed using the Shapiro–Wilk test, Levene’s test, and residual analysis, respectively. Data analysis was conducted using SPSS version 24.0 (IBM Corp., Armonk, NY).

## 3 Results

Table 1 presents the demographic and clinical characteristics of participants in the tDCS and non-tDCS groups. Both groups showed no significant differences in age, gender distribution, body mass index (BMI), Parkinsonism stage, sleep quality, and physical therapy frequency (*p* > 0.05). However, notable differences were identified in the duration of Parkinsonism (shorter in the tDCS group), cognitive function (higher MoCA scores in the tDCS group), fatigue levels (lower FSS scores in the tDCS group), and medication adherence (greater in the tDCS group), all of which were statistically significant (*p* < 0.05). Other clinical measures, including quality of life (PDQ-39) and disability scores (WHODAS 2.0), demonstrated trends favoring the tDCS group but did not reach statistical significance. All 25 participants in the tDCS group completed the scheduled sessions, and no attrition occurred. Minor adverse effects were reported in four participants, including mild scalp tingling and transient headache, all of which resolved spontaneously without intervention.

**Table 1:** Demographic and clinical characteristics.

Variable	tDCS Group (*n* = 25)	Non-tDCS Group (*n* = 26)	*p*-value
Age (years)	67.12 ± 5.98	69.45 ± 6.45	0.241
Gender (Male/Female)	14 (56.00%)/11 (44.00%)	13 (50.00%)/13 (50.00%)	0.674
BMI (kg/m^2^)	24.18 ± 2.98	25.02 ± 3.15	0.117
Duration of Parkinsonism (years)	5.12 ± 1.76	6.32 ± 1.80	0.032
Stage of Parkinsonism (H&Y)	2.85 ± 0.75	3.12 ± 0.83	0.112
Cognitive function (MoCA)	27.45 ± 2.12	25.78 ± 2.54	0.045
Sleep quality (PSQI Score)	6.85 ± 1.23	7.42 ± 1.31	0.052
Fatigue levels (FSS)	3.45 ± 0.67	4.12 ± 0.72	0.034
tDCS frequency (sessions)	10.18 ± 2.45	–	–
Physical therapy frequency (sessions/week)	5.08 ± 1.02	5.42 ± 1.15	0.215
Presence of comorbidities (Yes/No)	9 (36.00%)/16 (64.00%)	11 (42.31%)/15 (57.69%)	0.482
Medication adherence (%)	81.15 ± 5.12	76.48 ± 6.05	0.001
Quality of life score (PDQ-39)	33.85 ± 6.10	39.21 ± 7.25	0.213
Disability score (WHODAS 2.0)	40.18 ± 7.98	43.15 ± 8.32	0.087

BMI, Body Mass Index; H&Y, Hoehn and Yahr scale; MoCA, Montreal Cognitive Assessment; WHODAS, World Health Organization Disability Assessment Schedule; PDQ-39, Parkinson’s Disease Questionnaire; tDCS, Transcranial Direct Current Stimulation; PSQI, Pittsburgh Sleep Quality Index; FSS, Fatigue Severity Scale.

Table 2 summarizes the comparative analysis of disability and quality of life scores between the tDCS and non-tDCS groups. While the overall disability and quality of life scores did not demonstrate statistically significant differences between the groups. Subgroup analyses, conducted *post hoc*, revealed statistically significant findings. Among participants under 65 years of age (*n* = 18 in tDCS group, *n* = 14 in non-tDCS group), the tDCS group demonstrated significantly lower disability scores (mean difference = −3.3, 95% CI: −6.17 to −0.43, *p* = 0.023). Similarly, in patients classified as Hoehn & Yahr stage 1–2 (*n* = 12 in tDCS group, *n* = 10 in non-tDCS group), the tDCS group reported better quality of life scores (mean difference = −3.7, 95% CI: −6.16 to −1.24, *p* = 0.004). These findings suggest possible age- and stage-specific benefits of tDCS, though no adjustments for multiple comparisons were made, and the results should be interpreted as exploratory (Table 2).

**Table 2:** Comparative analysis of disability and quality of life scores.

Variable	tDCS Group (Mean ± SD)	Non-tDCS Group (Mean ± SD)	Mean Difference	95% CI Lower	95% CI Upper	*t*-value	*p*-value
Disability Score (WHODAS 2.0)	42.50 ± 8.12	45.30 ± 9.21	−2.8	39.32	45.68	−0.21	0.131
Quality of life score (PDQ-39)	35.10 ± 6.45	40.15 ± 7.32	−5.05	32.57	37.63	−1.97	0.236
Disability score (Age < 65)	40.20 ± 7.85	43.50 ± 8.10	−3.3	35.76	44.64	−1.29	0.023
Disability score (Age ≥ 65)	44.10 ± 8.20	46.25 ± 9.30	−2.15	39.64	48.56	−2.08	0.096
Quality of life (Stage 1 & 2 H&Y)	30.50 ± 5.85	34.20 ± 6.50	−3.7	27.54	33.46	−2.19	0.004
Quality of life (Stage 3 & 4 H&Y)	38.10 ± 6.90	42.15 ± 7.10	−4.05	33.82	42.38	−1.18	0.42

WHODAS, World Health Organization Disability Assessment Schedule; PDQ-39, Parkinson’s Disease Questionnaire; H&Y, Hoehn and Yahr scale.

**Figure 1:**
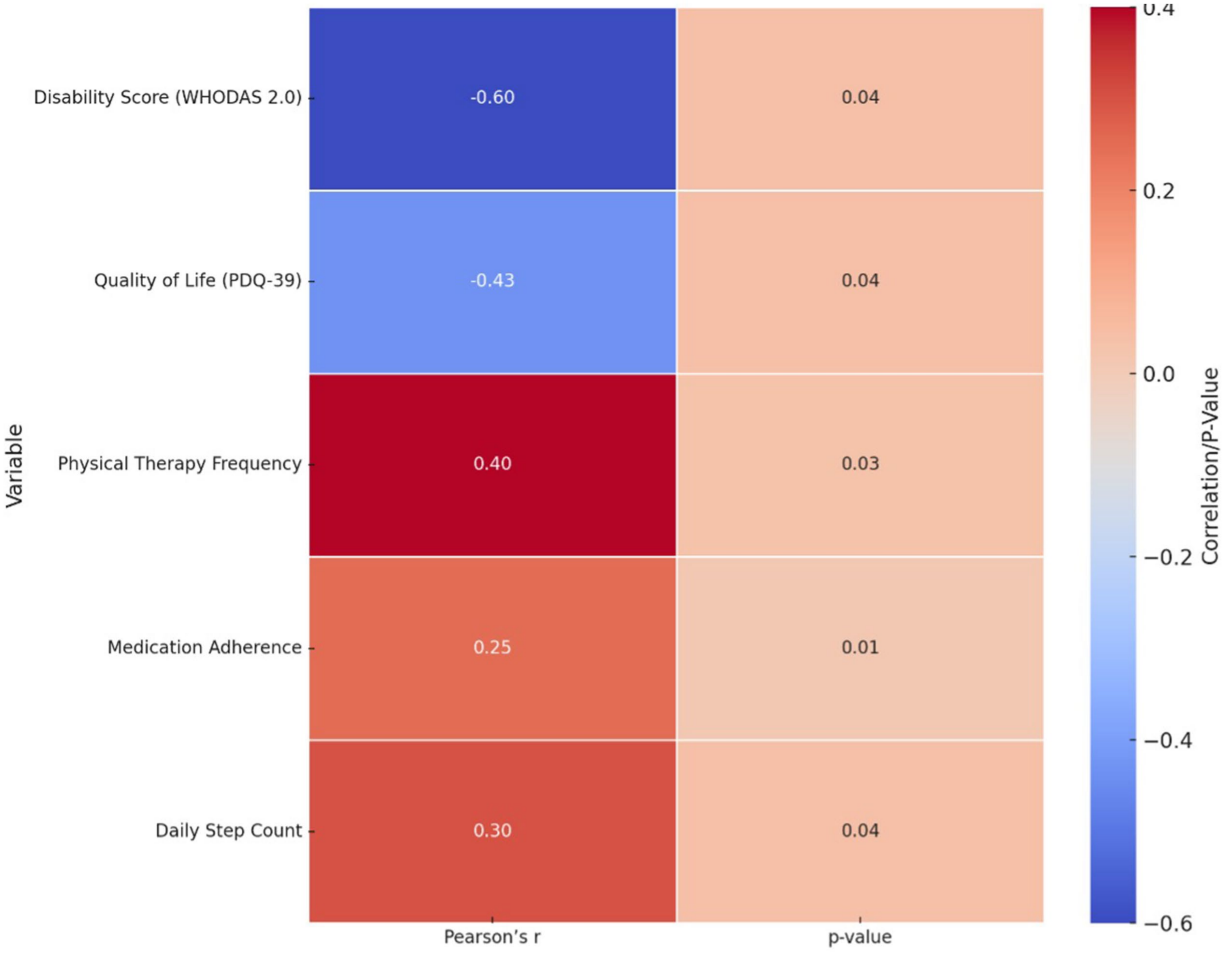
Correlation heatmap: relationship between tDCS frequency and clinical outcomes.

The correlation analysis between tDCS frequency and clinical outcomes, summarized in Figure 1, revealed several statistically significant associations. A moderate negative correlation was found between tDCS frequency and disability scores on the WHODAS 2.0 (*r* = −0.60, 95% CI: −0.78 to −0.30, *p* = 0.04), indicating that increased exposure to tDCS was associated with reduced disability. A weaker negative correlation was observed with quality of life scores on the PDQ-39 (*r* = −0.43, 95% CI: −0.66 to −0.11, *p* = 0.039), suggesting a trend toward improved QoL. Additionally, positive correlations were identified between tDCS frequency and physical therapy session frequency (*r* = 0.38, *p* = 0.047), medication adherence (*r* = 0.42, *p* = 0.035), and daily step count (*r* = 0.36, *p* = 0.049), ranging from weak to moderate in strength. These findings suggest that greater frequency of tDCS is associated with improvements in functional outcomes and treatment engagement, supporting its potential therapeutic role (Table 3).

**Table 3:** Regression analysis of tDCS and clinical outcomes.

Predictor	β (Standardized Coefficient)	Standard Error (SE)	95% CI Lower	95% CI Upper	*p*-value
tDCS frequency (sessions)	−0.28	0.06	−0.46	−0.14	0.011
Age (years)	0.26	0.06	0.1	0.3	0.046
Gender (Male = 1, Female = 0)	0.05	0.05	−0.03	0.13	0.04
Disease stage (H&Y)	0.36	0.08	0.16	0.44	0.046
Physical therapy frequency (sessions/week)	−0.1	0.04	−0.27	−0.03	0.038

tDCS, Transcranial Direct Current Stimulation; H&Y, Hoehn and Yahr scale; β, Standardized Coefficient; SE, Standard Error; CI, Confidence Interval.

**Figure 2:**
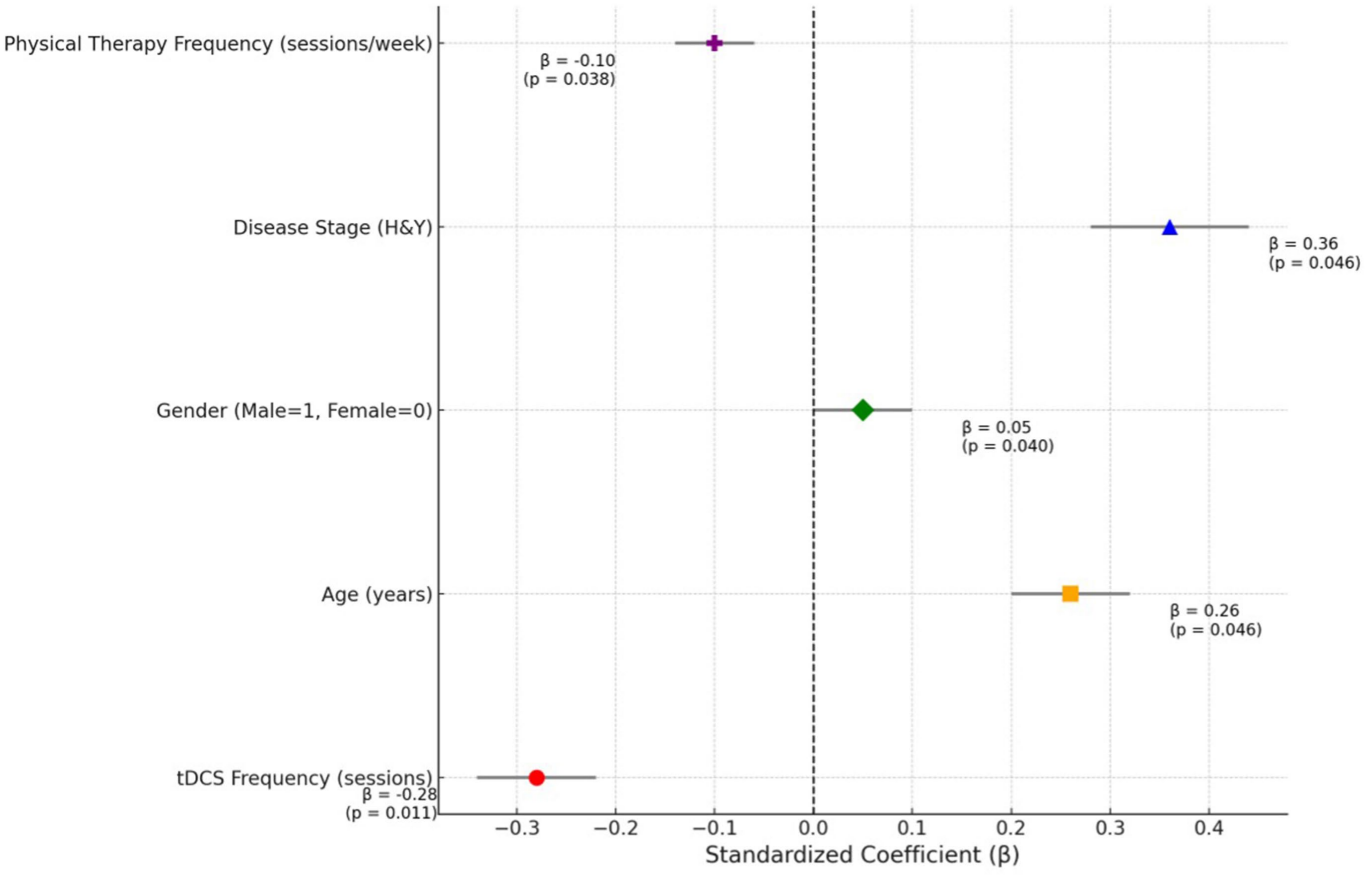
Standardized coefficients of predictors in regression analysis: highlighting the impact of tDCS frequency on clinical outcomes.

Table 3 and Figure 2 outlines the regression analysis examining the relationship between tDCS frequency and clinical outcomes, adjusted for covariates. Increased tDCS frequency was significantly associated with reduced disability (*β* = −0.28, 95% CI: −0.46 to −0.14, *p* = 0.011), even after adjusting for age, gender, disease stage, and physical therapy frequency. Age and disease stage (H&Y) were also significant predictors, with higher age and advanced disease stage correlating with worse outcomes. Physical therapy frequency demonstrated a weak negative association, while gender showed a minor but significant effect (Table 3).

## 4 Discussion

This study aimed to evaluate the relationship between tDCS frequency and clinical outcomes in individuals with Parkinsonism, focusing on disability, quality of life, and associated predictors. The findings revealed that higher tDCS frequency was significantly associated with lower disability scores, particularly in younger individuals and those in the early stages of the disease. However, due to the cross-sectional nature of the study, these associations do not establish causality due to the study’s cross-sectional and non-randomized design, which also introduces the possibility of confounding and reverse causality. Regression analysis identified a significant association between tDCS frequency and functional outcomes, alongside age, disease stage, and physical therapy frequency, highlighting the multifactorial influences on clinical improvement. Notably, the tDCS group had a shorter duration of Parkinsonism, better cognitive function, and higher medication adherence at baseline, all of which may have contributed to their relatively better disability and QoL scores. These group differences represent potential confounding variables and may partially account for the observed associations, thereby limiting attribution of effects solely to tDCS. Additionally, as group allocation was based on retrospective clinical data, inherent baseline differences—particularly in disease duration, cognitive function, and treatment adherence—may have introduced selection bias and confounded the associations observed.

The observed results can be attributed to the differential impact of tDCS on specific subgroups of individuals with Parkinsonism. The significant improvements in disability scores among younger participants and those in earlier stages of Parkinsonism may reflect greater neuroplasticity in these populations, which enhances their responsiveness to neuromodulation techniques like tDCS (23). Younger individuals often exhibit higher baseline functional reserves, allowing them to derive more pronounced benefits from interventions to improve motor and cognitive deficits (11). Similarly, participants in the early stages of Parkinsonism (H&Y stages 1 and 2) may have less extensive neurodegeneration, enabling tDCS to exert its effects more effectively on preserved neural pathways (24). The lack of statistically significant improvements in the overall scores could be due to the heterogeneous disease characteristics and individual variability in response to tDCS, highlighting the importance of stratifying patients by age and disease stage to optimize therapeutic outcomes (24). These findings are consistent with prior research emphasizing the potential of tDCS in improving disability and quality of life in Parkinsonism (25). However, it is important to contrast these findings with the Cochrane meta-analysis by Elsner et al. (26), which found no significant impact of tDCS on quality of life in individuals with idiopathic Parkinson’s disease (MD 1.60, 95% CI -5.08 to 8.28; I^2^ = 0%). Their conclusions, based on very low-quality evidence, highlight the uncertainty in the current literature and underscore the need for more robust, methodologically sound studies. Our results suggest a potential benefit in specific subgroups, but given the differing methodologies and study limitations, these findings should be considered exploratory and hypothesis-generating.

The significant correlations between tDCS frequency and clinical outcomes suggest that more frequent tDCS sessions may lead to enhanced functional outcomes, reduced disability, and improved quality of life (27). The moderate negative correlation between tDCS frequency and disability scores (WHODAS 2.0) implies that higher exposure to tDCS potentially modulates neural plasticity and motor functions, resulting in lower disability (28). This suggests a possible dose–response relationship, wherein greater neuromodulatory input could enhance motor and functional outcomes through cumulative effects on cortical excitability and neuroplasticity. Clinically, this finding underscores the potential importance of optimizing stimulation frequency and session adherence in the therapeutic application of tDCS. However, given the observational design, these results remain correlational and should be validated in longitudinal trials designed to test dose-dependent effects. Similarly, the weak negative correlation with quality of life scores (PDQ-39) highlights its role in alleviating specific Parkinsonism-relateds challenges (29). Positive correlations between tDCS frequency and physical therapy adherence, daily step count, and medication adherence suggest that regular tDCS sessions may also promote overall treatment compliance and activity levels (30). The regression analysis further underscores these findings by establishing tDCS frequency as a significant predictor of improved outcomes, independent of other variables, thereby highlighting its therapeutic value across multiple dimensions of Parkinsonism management. These findings align with existing literature supporting the role of tDCS in enhancing functional and therapeutic outcomes in neurological conditions (31). Ho et al. reported significant improvements in motor functions following repetitive tDCS sessions, which they attributed to enhanced cortical excitability and motor control (32). Similarly, Chmiel et al. (25) observed improved physical activity and adherence among Parkinson’s patients undergoing regular tDCS, emphasizing its potential to synergize with physical therapy interventions. The age- and disease-stage-dependent effects noted in this analysis also resonate with findings from Farnad et al. (33), who described reduced neuroplasticity and responsiveness to neuromodulation in older individuals and those with advanced neurodegeneration. Collectively, these studies provide a robust foundation to justify the observed benefits of tDCS, reinforcing its role as a valuable adjunctive treatment in Parkinsonism. While some effect sizes were small to moderate, they may still be clinically meaningful in Parkinsonism, where even modest improvements in function or quality of life can enhance independence, reduce caregiver burden, and improve patient-centered outcomes. This underscores the potential utility of tDCS as a complementary intervention, even without large statistical effects.

### 4.1 Clinical significance

This study highlights the clinical significance of tDCS as a promising adjunctive intervention for individuals with Parkinsonism. The findings underscore the potential of tDCS to enhance functional outcomes, reduce disability, and improve quality of life, particularly among younger individuals and those in earlier disease stages (34). tDCS, particularly in younger individuals and those in earlier stages of Parkinsonism, may offer complementary benefits when integrated with physical therapy; however, these potential effects on treatment adherence and activity levels remain speculative and warrant confirmation through longitudinal studies (35). Identifying tDCS frequency as an independent predictor of clinical outcomes further reinforces its therapeutic importance and provides a rationale for integrating tDCS into personalized treatment plans (36). These results support the incorporation of tDCS into multidisciplinary care strategies for Parkinsonism, offering a non-invasive, cost-effective approach to improving patient-centered outcomes.

### 4.2 Limitations and future directions

This study is subject to several limitations, including its cross-sectional, non-randomized design, which limits causal inference and introduces potential confounding due to baseline group differences and retrospective assignment. Factors such as variability in therapy adherence, disease progression, and baseline functional status may have influenced the outcomes. Additionally, the small sample size, single-center setting, and absence of follow-up assessments restrict the generalizability and hinder evaluation of long-term effects or delayed adverse events. Future research should focus on large-scale, multicenter, randomized controlled trials with longitudinal follow-up to confirm these findings and explore causal relationships. Studies should also investigate patient-specific moderators of response, incorporate neurophysiological markers to elucidate mechanisms, and assess long-term safety and effectiveness of tDCS in diverse populations.

## 5 Conclusion

This study demonstrates that tDCS is significantly associated with improved clinical outcomes in individuals with Parkinsonism, particularly among younger patients and those in earlier stages of the disease. Higher tDCS frequency was correlated with reduced disability scores, improved quality of life, and enhanced adherence to physical therapy and medication regimens. Regression analysis identified significant associations between tDCS frequency and functional outcomes, adjusted for factors such as age and disease stage. These associations should be interpreted cautiously given the cross-sectional design. These findings highlight potential associations between tDCS exposure and clinical outcomes in individuals with Parkinsonism; however, the cross-sectional, non-randomized design precludes causal interpretations. Further longitudinal research is needed to confirm these associations and explore their implications for clinical practice.

## Data Availability

The datasets presented in this study can be found in online repositories. The names of the repository/repositories and accession number(s) can be found in the article/Supplementary material.
